# Xiao-Xu-Ming Decoction Reduced Mitophagy Activation and Improved Mitochondrial Function in Cerebral Ischemia and Reperfusion Injury

**DOI:** 10.1155/2018/4147502

**Published:** 2018-06-19

**Authors:** Rui Lan, Yong Zhang, Tao Wu, Yun-Zhi Ma, Bao-Qi Wang, Hai-Zhong Zheng, Ya-Na Li, Yan Wang, Chun-Qing Gu, Ji-Tao Wu

**Affiliations:** ^1^Encephalopathy Hospital, The First Affiliated Hospital of Henan University of Chinese Medicine, Henan 450000, China; ^2^Cerebral Palsy Rehabilitation Department, The Third Affiliated Hospital of Zhengzhou University, Henan 450000, China; ^3^Henan University of Chinese Medicine, Henan 450000, China

## Abstract

We investigated whether Xiao-Xu-Ming decoction reduced mitophagy activation and kept mitochondrial function in cerebral ischemia-reperfusion injury. Rats were randomly divided into 5 groups: sham, ischemia and reperfusion (IR), IR plus XXMD (60 g/kg/day) (XXMD60), IR plus cyclosporin A (10 mg/kg/day) (CsA), and IR plus vehicle (Vehicle). Focal cerebral ischemia and reperfusion models were induced by middle cerebral artery occlusion (MCAO). Cerebral infarct areas were measured by triphenyl tetrazolium chloride staining. Cerebral ischemic injury was evaluated by hematoxylin and eosin staining (HE) and Nissl staining. Ultrastructural features of mitochondria and mitophagy in the penumbra of the ischemic cortex were observed by transmission electron microscopy. Mitophagy was detected by immunofluorescence labeled with LC3B and VDAC1. Autophagy lysosome formation was observed by immunofluorescence labeled with LC3B and Lamp1. The expression of LC3B, Beclin1, and Lamp1 was analyzed by Western blot. The rats subjected to MCAO showed worsened neurological score and cell ischemic damage. These were all significantly reversed by XXMD or CsA. Moreover, XXMD/CsA notably downregulated mitophagy and reduced the increase in LC3, Beclin1, and Lamp1 expression induced by cerebral ischemia and reperfusion. The findings demonstrated that XXMD exerted neuroprotective effect via downregulating LC3, Beclin1, Lamp1, and mitochondrial p62 expression level, thus leading to the inhibition of mitophagy.

## 1. Introduction

Mitochondria play crucial roles in energy production, programmed cell death, calcium homeostasis, reactive oxidative phosphorylation, and cell cycle regulation [1]. Autophagy is a vital conserved process for the bulk degradation and recycling of unnecessary proteins and organelle by the lysosomal degradation pathway. Starvation [2], hypoxia [3], and endoplasmic reticulum stress [4] notably activated autophagy. The roles of autophagy in the cerebral ischemia injury have been widely studied. Recent reports have found that autophagy [5–7] is widely evoked following cerebral ischemia-reperfusion injury. The findings have clarified that autophagy can be destructive [8] or protective [9]. Mitophagy, as one type of selective autophagy, is extremely important for maintaining mitochondrial homeostasis by removing damaged mitochondria [10]. Mitophagy which regulates the mitochondrial number in response to metabolic demand is a form of quality control to eliminate damaged mitochondria [11]. A large body of evidence has indicated the specific events containing endoplasmic reticulum (ER) stress, oxidative stress, and excitotoxicity involved in the cerebral ischemic brain initiate mitophagy [12]. Mitophagy is mainly evident in neurons [5] and astrocytes [13] subjected to cerebral ischemia. The role of mitophagy in cerebral ischemia is still controversial. Enhanced mitophagy attenuates mitochondrial dysfunction after cerebral ischemia [14, 15]. Other studies have suggested that deleterious autophagic processes lead to cell death and attenuated mitophagy may protect neurons from cerebral injury [16, 17]. It is generally accepted that failure to adequately remove damaged mitochondria or excessive degradation will lead to cell death [10].

Xiao-Xu-Ming decoction has been widely used to treat stoke and has notable effects [18]. Our previous studies have revealed that XXMD alleviates BBB disruption and protects neurovascular unit and mitochondria from cerebral injury induced by cerebral ischemia and reperfusion [19–21]. However, whether XXMD could regulate mitophagy following cerebral ischemia and reperfusion has not been studied yet. Therefore, the present study was aimed at exploring the effect of XXMD treatment on mitophagy and mitochondrial function after cerebral ischemia and reperfusion.

## 2. Methods and Materials

### 2.1. Animals

All experiments were performed on adult male Sprague-Dawley rats (Experimental Animal Center and Central Lab, Henan Province Hospital of Chinese Medicine, China) weighing 250~280 g. 92 rats were housed in temperature- and humidity-controlled (55 ± 5%) rooms with a 12 h light-dark cycle on a 12 : 12 h light. All animals were allowed to move and feed freely. The experimental protocols and animal handling procedures were approved by the Animal Care and Use Committee (ACUC) of the First Affiliated Hospital of Henan University of Chinese Medicine.

### 2.2. Preparation of XXMD

The crude drugs were purchased from Traditional Chinese Medicine Pharmacy of the First Affiliated Hospital of Henan University of Chinese Medicine. XXMD was prepared as previously reported [19–21]. The first decoction lasted for 1 h in a drug (1) : distilled water (10) ratio (*w* : *v*), and then the suspension was collected. The above process was repeated three times. The collected herbs were soaked with 75% ethanol for 24 h, and the liquid was gained. The above mixed suspension from three decoctions was centrifuged at 2000 ×g for 20 min, and the supernatant was obtained. Ethanol was slowly added to the suspension and kept stirred until the concentration reached 75% ethanol (*v*/*v*). Mixed suspension and the liquid obtained from the gruffs were centrifuged at 2000 ×g for 20 min and then concentrated at the final concentration of 2 g/ml (*w*/*v*). Eventually, the liquid was stored for the study.

### 2.3. Groups and Drug Administration

We previously examined the effects of different dosages of XXMD on cerebral ischemic injury and found that 60 g/kg/day gave maximal protective effects in the treatment of cerebral ischemic injury [19]. Previous studies have demonstrated that the immunosuppressor cyclosporin A (CsA, Solarbio Life Science, Beijing, China) reduces brain damage induced by cerebral ischemia in rats [22, 23]. It is well known that CsA could inhibit mitochondrial permeability transition, mitochondrial swelling [24, 25], and mitophagy [26, 27]. It has been reported that CsA suppresses cytochrome C efflux to the cytoplasm [28]. In the current study, we designed CsA as a control treatment in this study. In addition, evidence has determined that the dose of 10 mg/kg CsA is the optimal dose for neuroprotective effects in ischemic brain injury [28, 29]. In the study, rats were randomly divided into five groups: sham control (Sham) group, ischemia and reperfusion (IR) group, ischemia and reperfusion plus XXMD (60 g/kg/day, XXMD60) group, ischemia and reperfusion plus cyclosporin A (10 mg/kg, CsA) group, and ischemia and reperfusion plus dimethyl sulfoxide and ethanol (Vehicle) group.

The drug administrations were performed twice a day at 8:00 and 18:00 for 3 days before the induction of ischemia and lasted until the conclusion of the experiment. The rats in the XXMD60 group were orally administered XXMD. Others in the Sham and IR groups were given the same volume of distilled water. The rats in the CsA group were intraperitoneally injected with CsA, which was dissolved in polyethoxylated castor oil and 95% ethanol and further diluted with saline at a dosage of 10 mg/kg. The same solution lacking CsA was used for vehicle treatment.

### 2.4. Focal Cerebral Ischemia and Reperfusion Model

Focal cerebral ischemia and reperfusion was performed as described previously [19–21]. Briefly, the rats were anesthetized with 10% chloral hydrate (350 mg/kg, i.p.), and the left common carotid artery (CCA) was exposed. A unified nylon monofilament (Beijing Sunbio Biotech, Beijing, China), with a diameter of 0.26 mm and a length of 40 mm with its tip rounded, was inserted about 20 mm from the CCA bifurcation to block the origin of the middle cerebral artery (MCA). Reperfusion was initiated by withdrawal of the suture until the tip cleared the lumen of the ECA after 90 min of occlusion. In the current study, the rats in the IR, XXMD60, CsA, and Vehicle groups subjected to cerebral ischemia followed by reperfusion. Animals in the Sham group only underwent the same surgical procedure, but the suture was not inserted. Examination of neurological deficits was performed at 24 h after reperfusion. The neurological deficits were scored on a four-point scale described by Hara et al. [30], and the rats that underwent MCAO without any detectable neurological deficits were excluded from the further investigations.

### 2.5. Neurological Deficit Score

Examination of neurological deficits was performed at 24 h after reperfusion. The neurological deficits were scored on a four-point scale described by Hara et al. [30] with a minor modification: 0—no neurological deficit, 1—mild focal neurological deficit (animal showed forelimb flexion), 2—moderate focal neurological deficit (decreased resistance to lateral push and forelimb flexion without circling), and 3—severe focal deficit (decreased resistance to lateral push and forelimb flexion with circling). In the present study, the rats subjected to MCAO without any detectable neurological deficits were excluded from the following investigations and analyses to exclude operative failures.

### 2.6. Cerebral Infarct Area

The rats were sacrificed under deep anesthesia and perfused transcardially with normal saline. The brains were sectioned into coronal slices from rostral to caudal and stained with 1% 2,3,5-triphenyl tetrazolium chloride staining for 15 min at 37°C away from light. Then, the brain tissues were differentiated according to the white-colored infarct area and the red-purple noninfarct area. Cerebral infarct areas were calculated according to the previous study [20].

### 2.7. General Histology

The rats were anesthetized with 10% chloral hydrate (350 mg/kg, i.p.) and perfused transcardially with normal saline followed by 4% paraformaldehyde (0.1 M phosphate-buffered saline, pH 7.4) at 24 h after reperfusion. Then, the brain tissues were postfixed in the above fixative for 24 h and processed routinely for paraffin embedding. A series of 5 *μ*m thick sections were cut on a rotary microtome for hematoxylin and eosin (HE) staining and Nissl staining as previously described [20, 21]. And the number of intact cells in the penumbra of the ischemic cortex by Nissl staining was counted throughout five lesion regions randomly.

### 2.8. Electron Microscopy

Transmission electron microscopy was used to investigate the morphology of mitochondria and mitophagy after ischemia and reperfusion. At 24 h after reperfusion, the rats were perfused transcardially with cold 0.9% saline, followed by 4% paraformaldehyde and 1% glutaraldehyde. 1.0 mm^3^ of coronal sections located in peri-infarct areas of the ipsilateral cortex was postfixed in 2% osmium for 90 min, dehydrated, and embedded in Epon 812 Resin (TAAB, Berks, UK). Ultrathin (60 nm) sections were cut with a diamond knife, stained with uranyl acetate and lead citrate, and observed with an electron microscope (JEM-1400, Tokyo, Japan).

### 2.9. ATP Content Measurement

Purified mitochondria were extracted with a tissue mitochondrial isolation kit according to the manufacturer's instructions (Jiancheng Bioengineering Inc., Nanjing, China). Briefly, fresh brain cortical tissue was homogenized in 1 : 10 (*w*/*v*) ice-cold homogenization buffer plus 1 mM PMSF and centrifuged at 600*g* for 5 min at 4°C. The supernatants were centrifuged at 11,000*g* for 10 min at 4°C. The isolated mitochondrial fraction was collected for malonaldehydes (MDA, markers of oxidative damage), ATP assay, and Western blot. The ATP level was assessed with an ATP assay kit (Jiancheng Bioengineering Inc., Nanjing, China) according to the manufacturer's instructions and a previous study [14]. Mitochondrial fraction was centrifuged at 10,000*g* for 10 min, and then the supernatant was collected for the following assay. ATP reagents were added into a microwell for 5 min at 37°C, and samples were added and mixed for 10 s and measured by a Synergy HT multimode microplate reader. Results were expressed as nmol per mg protein calculated against a standard curve.

### 2.10. MDA Level Measurement

The MDA levels were examined with a MDA assay kit (Jiancheng Bioengineering Inc., Nanjing, China) according to the manufacturer's protocol and a previous study [14]. Briefly, the MDA reagent was incubated with the supernatant of mitochondrial lysis for 40 min at 95°C and then was detected with a Synergy HT multimode microplate reader at 532 nm absorbance. Results were expressed as nmol per mg protein.

### 2.11. Immunofluorescence Staining

The brain sections were dewaxed followed by the antigen repair method and then washed three times with PBS. The sections were blocked with 5% normal goat serum for 1 h at room temperature and incubated with a primary antibody at 4°C overnight. After washing with PBS, sections were incubated with an appropriate secondary antibody for 1 h at room temperature. Sections were stained with DAPI (Beyotime, Haimen, Jiangsu, China) for 15 min and observed with a fluorescence microscope (Olympus/BX51) after three 5 min washes with PBS. For double immunofluorescence, sections were incubated consecutively with pairs of primary and secondary antibodies. Sections were examined under a fluorescence microscope (Olympus/BX51, Tokyo, Japan). Double-stained cells of different groups were counted throughout five lesion regions randomly in the penumbra of the ischemic cortex.

### 2.12. Immunohistochemistry Staining

For immunohistochemistry, the brain slices were deparaffinized by xylene, the gradient alcohol was rehydrated, and the antigen was repaired. The slices were incubated with 0.3% H_2_O_2_ in PBS. After blocking with 5% normal goat serum, the sections were incubated with a mouse monoclonal Beclin1 antibody at appropriate concentration at 4°C overnight. After washing in PBS, the sections were incubated with the secondary antibody conjugated with horseradish peroxidase (Hua'an Biotechnology, Hangzhou, China) for 1 h at 37°C and then visualized using a general SP immunohistochemical kit (Solarbio Life Science, Beijing, China). The sections were photographed and observed with a light microscope (Olympus/BX51, Tokyo, Japan).

### 2.13. Western Blot

Equal qualities of protein solutions were separated by electrophoresis of appropriate concentrations of polyacrylamide gels and transferred to the polyvinylidene fluoride membranes (Millipore, Bedford, MA, USA). After blocking with 5% skim milk in Tris-buffered saline containing 0.1% Tween-20 (TBST) for 2 h at room temperature, the membranes were probed with primary antibodies at 4°C overnight. After washing with TBST three times for 10 min each, the membranes were incubated with the secondary antibody conjugated with horseradish peroxidase. Eventually, the targeted antigens were detected by standard chemical luminescence methods (Millipore, Bedford, MA, USA) on the Bio-Rad ChemiDoc™ MP imaging system. Band intensities were measured with Quantity One software (Bio-Rad Laboratories, Hercules, CA, USA).

### 2.14. Statistical Analysis

Data are presented as the means ± standard error of the mean (SEM). Statistical significance was determined by one-way ANOVA followed by Tukey's multiple comparison test or unpaired Student's *t*-tests using SPSS 11.5 for Windows (Chicago, IL, USA). All were considered statistically significant for *p* < 0.05.

## 3. Results

### 3.1. XXMD Reduced Cerebral Infarct Areas and Ameliorated Neurological Deficits

**Figure 1:**
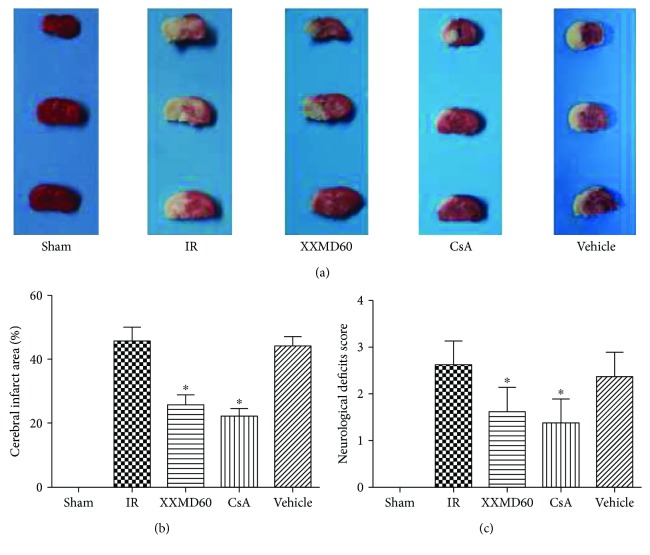
Cerebral infarct areas and neurological deficit score at 24 h after reperfusion. (a) Representative images of TTC-stained brain slices. The infarct area appeared white, whereas noninfarct areas were stained red-purple. (b) Quantitative analysis of cerebral infarct areas. (c) Effect of XXMD on the neurological deficit score. XXMD/CsA treatment significantly improved the neurological deficit score. Data are reported as the means ± SEM. *n* = 4 for TTC staining and *n* = 8 for the neurological deficit score. ^∗^*p* < 0.05 versus the IR group.

Cerebral infarct areas and neurological deficits induced by cerebral ischemia and reperfusion were evaluated by TTC staining and Hara's scores, respectively. The rats in the Sham group showed no neurological deficits and cerebral infarct areas. XXMD (60 g/kg/day) and CsA (10 mg/kg/day) treatment significantly reduced infarct areas in the territory of the middle cerebral artery compared to the IR group (*p* < 0.05). The rats subjected to ischemia and reperfusion showed severe neurological deficits; however, XXMD or CsA treatment significantly reduced neurological deficit scores compared with the IR group (*p* < 0.05) (Figure 1).

### 3.2. XXMD Alleviated Cell Injury

**Figure 2:**
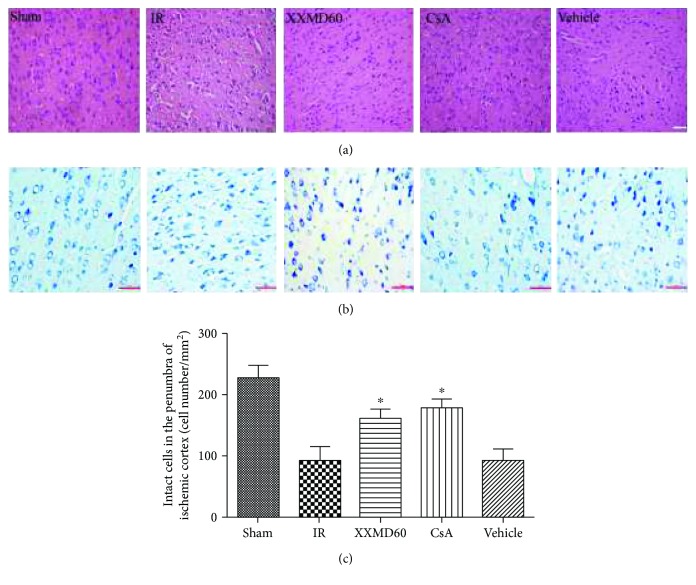
Histological changes of the penumbra of the ischemic cortex by groups. (a) HE images of cortical sections in different groups. (b) Representative images of Nissl staining in different groups. (c) Analysis of intact cells in the penumbra of the ischemic cortex. The number of intact cells in the XXMD60 group and CsA group was significantly higher than that in the IR group. Data are reported as the means ± SEM. *n* = 5; ^∗^*p* < 0.05 versus the IR group. Scale bar = 50 *μ*m.

Cell injury was estimated by HE staining and Nissl staining. The images of HE staining showed that the cells with the abundant cytoplasm and clear nuclei were arranged orderly in the Sham group. After 90 min of ischemia and 24 h of reperfusion, most neurons in the ischemic penumbra of the cerebral cortex appeared shrunken and deep stained in the IR group. In contrast to the IR group, residual neuron structures were improved with visible membranes and nuclei and more intact neurons in the XXMD60 and CsA groups. The characteristics of ischemic injury in the Vehicle group were similar to those in the IR group. Nissl staining found that most cells were shrunk with an enlarged intercellular space and had deep color staining in the IR and Vehicle group. However, these characteristic changes were improved by XXMD and CsA treatment. Furthermore, there were more intact cells in the penumbra of the ischemic cortex in the XXMD-treated or CsA-treated rats compared to the IR group (*p* < 0.05) (Figure 2).

### 3.3. XXMD Reduced the Formation of Mitophagy in the Ischemic Cortex Penumbra

**Figure 3:**
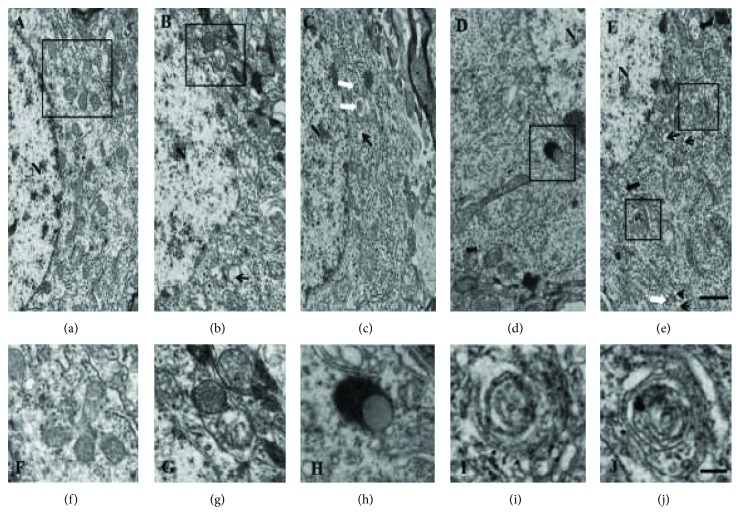
Representative electron microphotographs of neuronal mitophagy in the ischemic penumbra of the cerebral cortex at 24 h after the reperfusion. (a) The Sham group with normal mitochondrial ultrastructures. (f) Enlargement of the boxed area in (a). (b) Typical mitophagy structure and swollen mitochondria were revealed in the IR group. (c) Autophagy structure (white arrow) and autophagic vacuoles (small black arrow) were also shown in the XXMD60 group. (d) Autolysosomes were observed in neurons in the CsA group. High magnifications revealed autolysosome (h). (e) The characteristics of autophagy structure (white arrow), autophagic vacuoles (small black arrow), and swollen mitochondria (big black arrow) were observed in the Vehicle group. High magnifications revealed typical mitophagy structure (i and j). N: nuclear, *n* = 3, scale bar = 1 *μ*m (a–e), scale bar = 0.5 *μ*m (f–j).

The characteristics of neuronal mitophagy in the ischemic penumbra were observed by electron microscopy. Healthy mitochondria and nuclei were observed in the Sham group (Figure 3(a)). After 24 h of reperfusion, neurons displayed mitochondrial swelling, loss of matrix density, and the formation of vacuoles. Some partially degraded mitochondria and numerous autophagic vacuoles were detected in the IR group and Vehicle group (Figures 3(b) and 3(e)). A higher magnification showed double-membraned autophagosomes containing damaged mitochondria (Figures 3(g), 3(i), and 3(j)). In contrast, mitochondria with some slight swelling and nearly normal matrix density were detected in the XXMD-treated group and CsA group (Figures 3(c) and 3(d)). Autophagosomes were frequently observed and were more abundant than in the IR and Vehicle groups. Typical autolysosomes were observed in neurons in the CsA group (Figure 3(h)).

### 3.4. XXMD Improved Mitochondrial Function

**Figure 4:**
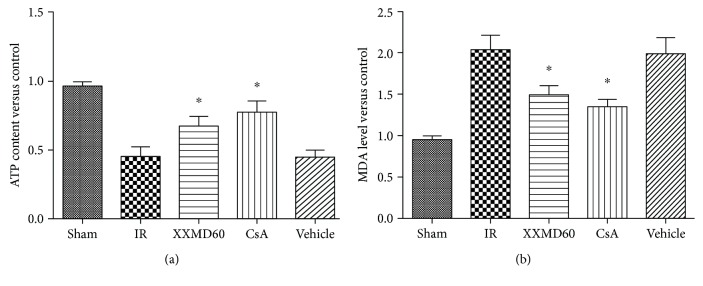
XXMD improved mitochondrial function at 24 h after reperfusion. (a) ATP content of different groups. (b) MDA levels of different groups. Data are reported as the means ± SEM. *n* = 5, ^∗^*p* < 0.05 versus the IR group.

Levels of ATP and MDA were determined to assess mitochondrial function after 24 h of reperfusion. MDA levels in the IR and Vehicle groups were significantly increased compared with those in the sham rats (*p* < 0.05), while treatment with 60 g/kg/day XXMD or 10 mg/kg/day CsA dramatically suppressed the production of MDA in the rats subjected to cerebral ischemia compared with that in the IR group (*p* < 0.05). In addition, we found the significant decrease in the ATP level after cerebral ischemia and reperfusion (*p* < 0.05), and XXMD or CsA treatment notably prevented the decrease in ATP levels compared with the IR group (*p* < 0.05) (Figure 4).

### 3.5. XXMD Downregulated Mitophagy in the Penumbra of the Ischemic Cortex

**Figure 5:**
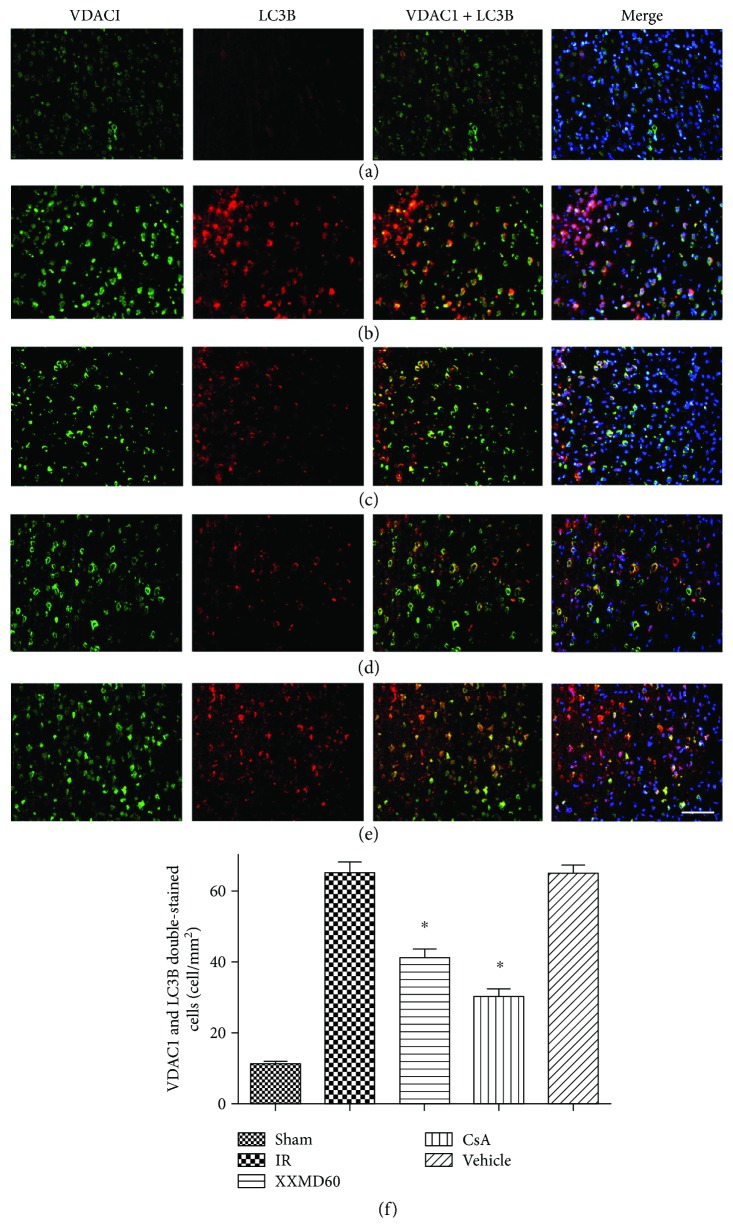
Double immunofluorescence staining for VDAC1 and LC3B in the penumbra of the ischemic cortex at 24 h after reperfusion. VDAC1 and LC3B double-stained cells (yellow) indicated mitophagy. (a) Representative microphotographs of rats in the Sham group. (b) Representative microphotographs of rats in the IR group. (c) Representative microphotographs of rats in the XXMD60 group. (d) Representative microphotographs of rats in the CsA group. (e) Representative microphotographs of rats in the Vehicle group. The images indicated that LC3B-positive cells were significantly detected in the peri-infarct area. Overlapping images showed that LC3B partly colocalized with VDAC1. (f) Quantification of VDAC1 and LC3B double-stained cells. *n* = 4, scale bar = 50 *μ*m. ^∗^*p* < 0.05 versus the IR group.

Immunofluorescent staining of microtubule-associated protein 1 light chain 3B (LC3B, autophagic marker) and voltage-dependent anion-selective channel 1 (VDAC1, mitochondrial marker) was used to determine mitophagy. The nuclei were stained by 4′,6-diamidino-2-phenylindole (DAPI). The immunoreactive products for LC3B were distributed in the cytoplasm. In sham-operated animals, cortical cells displayed diffuse and weak staining for LC3B in the cytoplasm. The morphology of positive cells with the shrunken cytoplasm was changed following cerebral ischemia and reperfusion in the IR and Vehicle groups. After being irritated by this stimulus, intense LC3B staining was mainly observed in the ischemic penumbra and appeared granular in the cytoplasm. LC3B (red) that colocalized with VDAC1 (green) in the cytoplasm showed that increased mitophagy (yellow) occurred in cortical neurons, suggesting that ischemia and reperfusion significantly promoted mitophagy (*p* < 0.05). Immunointensity of mitophagy was reduced in samples from the XXMD-treated or CsA-treated groups, and the inhibitory effect of CsA treatment on mitophagy was more obvious (*p* < 0.05) (Figure 5).

### 3.6. XXMD Reduced Autolysosome Formation in the Penumbra of the Ischemic Cortex

**Figure 6:**
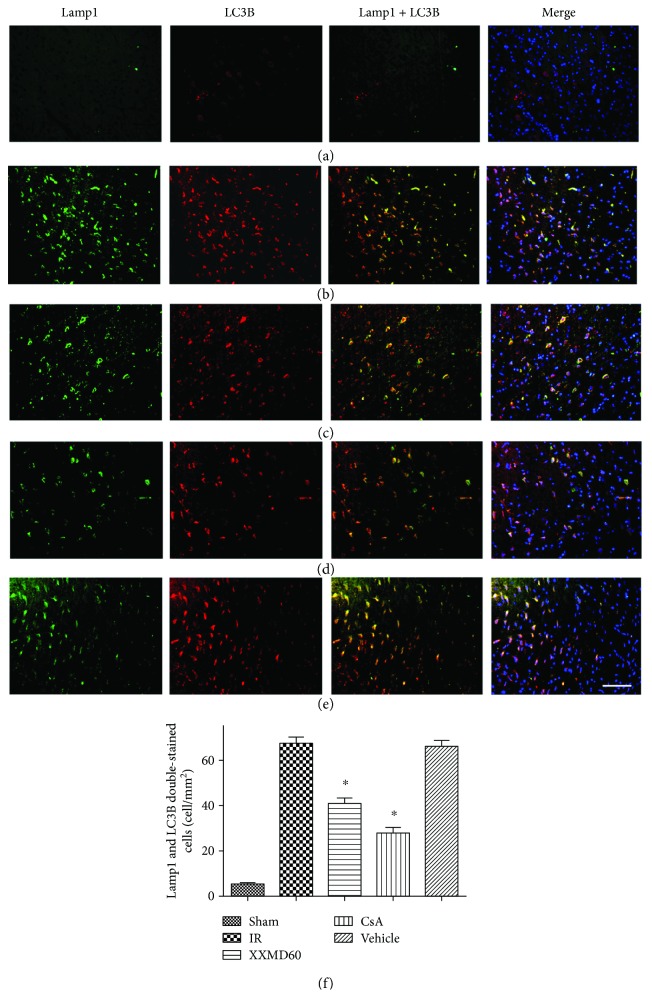
Double immunofluorescence staining for LC3B and Lamp1 of different groups in the penumbra of the ischemic cortex at 24 h after reperfusion. LC3B (red) immunostaining that colocalized in Lamp1-positive cells (green) indicating autophagic lysosomes. (a) Representative microphotographs of rats in the Sham group. (b) Representative microphotographs of rats in the IR group. (c) Representative microphotographs of rats in the XXMD60 group. (d) Representative microphotographs of rats in the CsA group. (e) Representative microphotographs of rats in the Vehicle group. (f) Quantification of Lamp1 and LC3B double-stained cells. *n* = 4, scale bar = 50 *μ*m. ^∗^*p* < 0.05 versus the IR group.

Autophagosomes fused with lysosomes and formed autolysosomes to degrade proteins and organelles and then complete the course of autophagy. Immunofluorescent staining of LC3B (autophagic marker) and lysosomal-associated membrane protein 1 (Lamp1, lysosome marker) was used to determine autolysosome formation in different groups. The positive cells labeled with Lamp1 were notably increased in the rats subjected to MCAO compared with the rats in the Sham group (*p* < 0.05). LC3B (red) that colocalized with Lamp1 (green) in the cytoplasm showed autophagy lysosomes, and the images suggested that ischemia and reperfusion activated the significant increases in autophagy lysosome formation (*p* < 0.05). However, the increase was sharply suppressed in samples from the XXMD-treated or CsA-treated groups (*p* < 0.05). Additionally, the greatest lower effect on autophagy lysosome formation was exerted by CsA (*p* < 0.05) (Figure 6).

### 3.7. XXMD Regulated Mitophagy-Related Protein Expression and p62 Translocation to the Mitochondria

**Figure 7:**
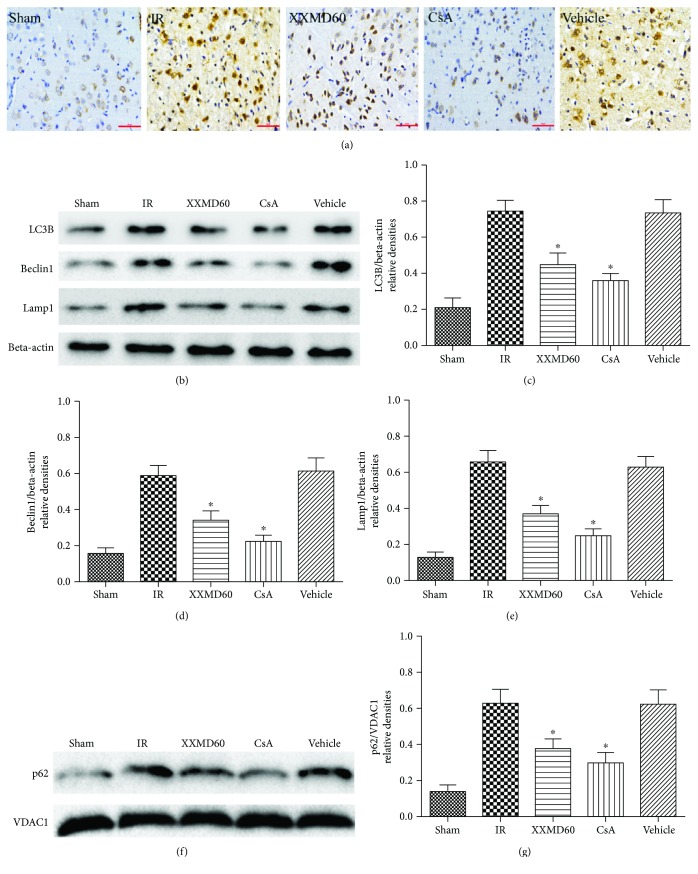
Beclin1 immunohistochemistry and expression levels of LC3, Beclin1, Lamp1, and mitochondrial p62 at 24 h after reperfusion following 90 min of cerebral ischemia. (a) Representative microphotographs of immunohistochemistry for Beclin1. (b) Western blots of LC3, Beclin1, and Lamp1. Beta-actin bands were the internal control. (c) Quantitative evaluation of LC3B. (d) Quantitative evaluation of Beclin1. (e) Quantitative evaluation of Lamp1. (f) Western blot of p62 in the mitochondrial fraction. VDAC1 bands were the internal control. (g) Quantitative evaluation of mitochondrial p62. Data are reported as the means ± SEM. *n* = 4, ^∗^*p* < 0.05 versus the IR group.

To investigate the effects of XXMD on mitophagy and the activity of the lysosomal pathway, we detected expression of mitophagy-related proteins including LC3B, Beclin1, and Lamp1 in the ischemic cortex 24 h after reperfusion by Western blot and immunohistochemistry. The results of Western blots were consistent with immunohistochemistry staining for Beclin1 and immunofluorescence staining for LC3B and Lamp1. The findings showed that cerebral ischemia and reperfusion resulted in a significant increase in LC3B, Beclin1, and Lamp1 expression compared with the Sham group (*p* < 0.05). Treatment with XXMD or CsA markedly downregulated LC3B, Beclin1, and Lamp1 expression after 90 min of ischemia and 24 h of reperfusion compared with the IR group (*p* < 0.05). p62 can bind to ubiquitinated mitochondria to direct phagophores through a LC3-binding domain [14, 31, 32]. We further examined p62 expression levels in mitochondrial fractions by Western blot. As shown in Figures 7(f) and 7(g), a significant increase in p62 expression was detected in the mitochondrial fractions after ischemia and reperfusion in contrast to the rats in the Sham group (*p* < 0.05). These results also showed that XXMD/CsA treatment reduced p62 translocation to mitochondria, demonstrating that XXMD/CsA significantly decreased mitophagy (*p* < 0.05) (Figure 7).

## 4. Discussion

In present study, the rats treated with XXMD/CsA showed markedly decreased brain damage at 24 h after reperfusion following 90 min of cerebral ischemia. XXMD treatment significantly suppressed mitophagy activation, and reduced mitophagy may play a vital role in brain injury following ischemia and reperfusion.

Cerebral ischemia and reperfusion injury is a complex pathological process. Necrosis, apoptosis, and autophagic cell death, as three different types of cell death, are considered to involve in neural tissue damage [33]. Plenty of evidence has indicated that mitophagy, characterized by abnormal mitochondria within autophagosomes or fused with autophagic vesicles in the ischemic penumbra area, is activated [14, 34]. Insufficient or excessive mitophagy can result in cellular death, caused by damaged mitochondrial accumulation or removal of indispensable mitochondria, respectively [17, 35]. Our previous findings have demonstrated that XXMD could protect mitochondria from ischemic injury and inhibit apoptosis through the mitochondrial p53 pathway [21]. However, whether XXMD involves in modulating mitophagy in brain injury was unknown. In this study, we further explored mitophagy induced by cerebral ischemia and reperfusion and the effects of XXMD treatment on mitophagy and mitochondrial function after cerebral ischemia and reperfusion. Electron microscopy showed that swollen or vacuolated mitochondria with fragmented cristae and activated mitophagy appeared following ischemia and reperfusion. Nevertheless, mitochondrial damage was alleviated indicating relative integrity of cristae structure, and a few structures of autophagy and mitophagy appeared in the XXMD- or CsA-treated group. Mitochondria are those essential organelles for generation and action of reactive oxygen species (ROS) in brain cells [36]. Damaged mitochondria following ischemia and reperfusion could result in the increase in ROS, and meanwhile, mitochondrial ATP levels are considered an indicator of cell death [14]. The present results demonstrated that treatment with 60 g/kg/day XXMD or 10 mg/kg CsA dramatically prevented the decrease in ATP content and reduced MDA levels, suggesting that inhibition in ROS production and protection of mitochondrial function may also have a role in protective effects of XXMD.

LC3B, the most widely used marker for revealing the presence of autophagosomes during autophagy activation, is attached to the autophagosome membrane, suggesting that the amount of LC3B closely correlates with the number of autophagosome [36, 37]. The results showed that increased LC3B immunoreactivity was mainly located in the cytoplasm of cells in the penumbra of the ischemic cortex and XXMD treatment downregulated LC3B expression. VDAC1, the abundant mitochondrial outer membrane protein, forms a cylindrical channel [38]. Moreover, we detected mitophagy using double immunofluorescence labeling with anti-LC3B and VDAC1 antibodies. LC3B colocalized with VDAC1 in the cytoplasm, suggesting that ischemia and reperfusion promoted significantly mitophagy. There is a significant decrease in LC3B/VDAC1 colabeling in the ipsilateral hemisphere in XXMD-treated and CsA-treated groups. Beclin1, a component of the class 3 phosphoinositide 3-kinase (PI3K) complex, is required for autophagy and LC3, and Beclin1 is involved in the early stages of autophagosome formation [33, 36]. The data showed that LC3B and Beclin1 expression was increased after 24 h of reperfusion, whereas XXMD or CsA treatment robustly prevented ischemia-reperfusion-induced upregulation of LC3B and Beclin1 expression. Additionally, marked upregulation of the lysosomal marker Lamp1 further supported the notion that mitophagic/lysosomal pathway was activated in cerebral ischemia and reperfusion. XXMD or CsA treatment downregulated Lamp1 expression. In addition, p62, a sensitive marker for mitophagy, recruits phagophores through an LC3-binding domain to degrade the damaged mitochondria [31, 32]. We further demonstrated that XXMD downregulated p62 translocation to mitochondria in response to ischemia-reperfusion injury. Based on the above findings, we hypothesized that XXMD modulated selective mitochondrial autophagy formation in the penumbra of the ischemic cortex following ischemia and reperfusion in the rat brain.

The evidence presented in this study indicated that XXMD treatment may exert protective effects on cerebral ischemia-reperfusion injury partly by the regulating mitophagy. This may shed lights on a novel mechanism of XXMD treatment against focal cerebral ischemia and reperfusion injury.

## 5. Conclusion

In conclusion, the present study demonstrated that mitophagy was increased in the penumbra of the ischemic cortex after cerebral ischemia and reperfusion. Additionally, XXMD/CsA downregulated LC3B, Beclin1, and Lamp1 expression and reduced p62 translocation to mitochondria, which may be related to attenuated mitochondrial dysfunction and suppressed mitophagy after stroke.

## Data Availability

The data used to support the findings of this study are available from the corresponding author upon request.
